# 

**Published:** 1971-10

**Authors:** 


The
Bristol Medico-Chirurgical Journal
(Incorporating the Medical Journal of the South-West)
Established 1883

Vol. 86 (iv) OCTOBER 1971 No. 320

### BRISTOL MEDICO-CHIRURGICAL JOURNAL

Editor: Dr. N. J. Brown, M.B., F.R.C.P., F.R.C.Path.

Published Quarterly

Annual Subscription 80p (16/-) Post Free

### BRISTOL MEDICO-CHIRURGICAL SOCIETY

**President 1971-72 : Dr. C. C. Morgans**

**President-elect : Dr. J. Macrae**

**Honorary Secretary : Dr. I. S. Bailey, 7 Percival Road Bristol 8.**

**Honorary Treasurer : Dr. Frank Ross, Bristol Royal Infirmary, Bristol 2.**

The Society was founded in 1874. In 1883 publication of the Journal began and has continued quarterly ever since then. During the years 1949 to 1962 it appeared under the title of "The Medical Journal of the South West".

### THE BRISTOL MEDICO-CHIRURGICAL JOURNAL

**Editorial Committee**

Editor — Dr. N. J. Brown, Southmead Hospital, Bristol.

Members — Mr. J. B. M. Roberts.
Dr. M. B. Lennard.
Professor R. C. Wofinden.
Dr. D. W. Wright.
The Hon. Treasurer and Hon. Secretary of the Society.

Business Manager — Dr. P. J. F. Baskett, Frenchay Hospital, Bristol.

Secretary — Mr. B. P. Jones, The Library, Medical School, University Walk, Bristol BS8 1TD.

### NOTICE TO CONTRIBUTORS

The Journal is especially concerned to provide a place for the recording of medical thought, interests, and practice in and around Bristol. It therefore welcomes articles that, as well as being of immediate interest to members, will document the local and contemporary medical scene for our successors.

Original articles are invited provided that they have not been published elsewhere. References in the text should be by author's name, with year of publication in parentheses; they should be listed at the end in alphabetical order, the name of each journal or book being given in full — not abbreviated — and the title of the article added. Illustrations should be supplied ready for reproduction. Line drawings should be in Indian ink on firm white paper or board. The author will be informed if it is found necessary to ask him to pay for the making of blocks.

The following contributions will be especially welcome : notes of forthcoming events, meetings held, degrees conferred, staff appointments, honours and public appointments and other local news.

Twenty-five reprints of his article will be supplied free to each contributor. Quotations for larger quantities will be sent out with proofs and must be ordered when the corrected proofs are returned.

### Bristol Medico-Chirurgical Society

### PROGRAMME FOR 1971-72

**13th October, 1971**
Annual General Meeting and President's Address.

**10th November, 1971**
Clinical Meeting, Stoke Park Hospital.

**8th December, 1971**
Dr. P. S. Byrne, Director, Department of General Practice, Manchester University. The Hard Times of General Practice.

**12th January, 1972**
Dr. C. P. de Fonseka. Accidents in the Home.

**9th February, 1972**
Dr. W. Lumsden Walker. Crises of Adolescence.

**8th March, 1972**
Mr. A. G. McPherson. Some Surgical Problems of the New Born.

**12th April, 1972**
Prof. M. A. Epstein. Some Problems relating to Suspected Human Tumour Virus.

**10th May, 1972**
Mr. Crispin Gill, Editor, The Countryman.

### Bristol Medico-Chirurgical Society

At a meeting in March 1971 the Society decided to seek Registration under The Charities Act 1960. This made necessary certain alterations in the Laws (henceforth to be known as the Rules) of the Society. These alterations, together with changes in the subscription were approved at the same meeting.

The Society has now been duly registered as a Charity and the revised Rules are as set out below.

### RULES OF THE SOCIETY

1—(a) The Society shall be called "The Bristol Medico-Chirurgical Society."
(b) The object of the Society shall be the advancement of the Art and Science of Medicine in all its branches.

2—The Society shall consist of—
(a) A President, President elect, Honorary Secretary, Honorary Medical Librarian, Honorary Treasurer, the Editor and Assistant Editor of the Bristol Medico-Chirurgical Journal, who ex-officio, together with nine elected members, shall form the Committee.
(b) Honorary Members, not exceeding ten in number.
(c) Ordinary Members.

3—(a) The President shall be elected annually, by ballot if demanded, at the Annual Meeting of the year before his year of office. He shall not be eligible for election two years in succession.
(b) The Honorary Secretary, Honorary Medical Librarian, and Honorary Treasurer shall be elected for one year at the Annual Meeting, by ballot if demanded, and shall be eligible for re-election.
(c) At the Annual Meeting in each year one third of the nine elected members of Committee shall retire in rotation and shall not be eligible for re-election for the ensuing year.
(d) Before the 20th day of September in each year, the names of candidates for election to the vacancies on the Committee shall be sent in writing to the Honorary Secretary, each name shall be proposed and seconded by two members who shall sign the proposal paper. Additional nominations may be made at the Annual Meeting.
(e) Each member of the Society at the Annual Meeting may vote for as many candidates as there are vacancies, but not fewer, the voting shall be by ballot.
(f) Those candidates securing the greatest number of votes shall be elected. In the case of a tie, the President shall have a casting vote.
(g) The Committee shall fill any casual vacancy in their number, the member so elected retiring at the time when the member whom he has replaced would otherwise have retired.

4—"Proposal to Elect an Honorary Member" shall be notified in those words to the Committee when convening it. If all those present agree to the proposal, and not otherwise, the Secretary shall in like manner give notice to all Members before the next meeting of the Society, at which the proposal shall put from the Chair: voting shall be as for the election of ordinary Members.

5—Any Registered Medical Practitioner desirous of entering the Society shall be proposed and seconded by Members at one meeting, and shall be voted for by show of hands at the following meeting—one adverse vote in five to exclude. The name, residence, and professional qualifications of any Candidate for Membership, together with the names of the proposer and seconder, shall be announced in the notice convening the meeting at which the election is to take place.

*Note—A former member of the Society may rejoin with the approval of the Committee, without re-election.*

6—If the conduct of any Member be deemed prejudicial to the welfare of the Society, the Secretary, either by instruction of the Committee or on a requisition signed by not less than ten Members, shall bring the matter under the consideration of the Society at the next meeting or the next but one—provided always that at least fourteen clear days' notice of the meeting shall be given to each Member of the Society, the notice stating that a matter arising under Rule 6 is to be considered and provided that the Member whose conduct is in question shall be so informed and be given the opportunity of explaining his conduct either in writing or personally to the meeting. If no explanation be given or if given not considered satisfactory, and on a show of hands, it be so voted by a majority of two-thirds of the Members present, the Member shall be expelled.

7—A Meeting shall be held on the second Wednesday in each month, from October to May inclusive at the time arranged by the Committee. The meeting in October shall be the Annual meeting, at which the election of Officers and Committee shall take place.

8—The Subscription to the Society shall be £4 per annum, payable in advance. This sum shall include the subscription to the Journal and the use of the Library, with the privileges appertaining to the circulation of Library books. The subscription to the Journal to non-members of the Society shall be £2 per annum.

9—Any Member may introduce a Visitor, with the permission of the President.

10—The following shall be the order of business:—
- (a) The minutes of the preceding Meeting to be read and confirmed.
- (b) New Members to be voted for.
- (c) Any candidates for Membership to be proposed and seconded.
- (d) Any miscellaneous business permitted by the President.
- (e) Remarks upon patients shown.
- (f) Remarks upon specimens exhibited.
- (g) Papers to be read and discussed.

11—The President shall be Chairman and shall have unlimited authority on all points of order, and, in addition to his vote as Member, be entitled to a casting vote. In the absence of the President, the members shall elect a chairman for that meeting, during which he shall have all the privileges and powers of the President.

12—The Secretary shall give notice of Meeting to all the Members; shall keep an accurate record of the proceedings of the Society and of the Committee in the books kept for the purpose, and shall furnish a report at each Annual Meeting.

13—The Treasurer shall receive subscriptions and pay all debts incurred by the Society and shall furnish a report of the financial position of the Society and the Journal at each Annual Meeting.

14—The Committee shall assemble for business as often as any special matter may require it—five Members constituting a quorum. They shall have the control of all the funds of the Society, and they shall make such regulations as the welfare of the Society shall demand.

15—The Society shall publish a Journal called "The Bristol Medico-Chirurgical Journal", directed by an Editor and Editorial Committee appointed by the Committee of the Society.

16—The Society shall appoint three of its Members to represent the Society upon the University Library Sub-Committee: one of these Members shall be the Honorary Medical Librarian.

17—Two auditors shall be appointed at the Meeting of the Society in May, in order that the accounts may be audited before the Annual Meeting.

18—(a) The Rules of the Society shall be printed and a copy sent to each member.
- (b) No alteration to any rule shall be made until it has been notified in the circular convening a meeting, in its approved form, carried at that meeting and confirmed at the succeeding meeting.
- (c) No alteration shall be made in any rule which shall have the effect of causing the Society to cease to be a charity in law.

19—The Editor of the Journal of the Society shall have the prior right to publish any communication made at any meeting of the Society, except that cases and papers which are intended for other Journals may be exhibited or read with the permission of the President of the Society, on the understanding that they are not to be at the disposal of the Journal Committee.

20—In the event of the Society being dissolved and its affairs wound up, after the payment of all its outstanding debts and liabilities, its assets will pass to another charity which has similar objects to those of the Society.

21—No alteration shall be made in Rule 1b, 18c or 20 without the approval of the Charity Commissioners.

### JOURNAL BYE-LAWS AND REGULATIONS

1—The management of the Journal shall be in the hands of the Committee of the Society.

2—The Editor shall be appointed by the Committee of the Society and shall hold office during the pleasure of the Committee. He shall have full control of the Journal, the admission or rejection of articles, notes, reviews or advertisements.

3—The Assistant Editor shall be appointed by the Committee on the nomination of the Editor. He shall hold office during the pleasure of the Committee and shall assist in the literary and secretarial management of the Journal.

4—The Treasurer of the Society shall undertake the Business management of the Journal.

5—The Editorial Committee shall consist of: The Editor, The Assistant Editor and the Treasurer of the Society; together with not more than seven members of the Society appointed annually by the Committee of the Society.
It shall have power to co-opt.

6—The Journal shall be delivered free to Subscribers, and also to Members of the Society whose subscriptions are not in arrear.

7—The Secretary of the Society shall summon a Meeting of the Committee of the Society upon the request of either the Editor or the Assistant Editor.

8—The name of any subscriber to the Journal whose subscription is in arrear for one year shall be removed from the list of subscribers, without prejudice to the right to recover subscriptions due and unpaid.

9—The Journal shall be published quarterly in January, April, July and October.

10—All illustrations shall be paid for by the Authors of Papers, but the Editorial Committee shall have power to pay for such illustrations from the funds of the Journal.

11—All exchange Journals and Books received through the Society's Journal shall be placed in the University Medical Library.

### The Medical Library

In 1890 the Bristol Medico-Chirurgical Society founded a medical library which in 1892 was combined with that of the Bristol Medical School. This became the University of Bristol Medical Library in 1925 and an agreement in that year confirmed the privilege of Members of the Society to use the University Medical Library.

### A Selection of Recent Additions to the Medical Library

**ACKERMAN, L. V.** and **DEL REGATO, J. A.:** Cancer: diagnosis, treatment and prognosis. Ed. 4. (Mosby)

**AGATE, J.:** Practice of geriatrics. Ed. 2. (Heinemann)

**ANDERSON, W. F.:** Practical management of the elderly. Ed. 2. (Blackwell)

**BETHLEM, J.:** Muscle pathology. (North-Holland)

**BEVAN, J. M.** and **DRAPER, G. J.:** Appointment systems in general practice. (Oxford Univ. Press)

**BLAIR, J. E.** et al.: Manual of clinical microbiology. (Williams & Wilkins/Livingstone)

**BLANDY, J. P.** et al.: Tumours of the testicle. (Heinemann)

**BREWER, G. J.** ed.: Red cell metabolism and function. (Plenum Press)

**BRIM, O. G.** et al., eds.: The dying patient. (Russell Sage Foundation/Basic Books)

**BROWN, R. J. K.** and **VALMAN, H. B.:** Practical neonatal paediatrics. (Blackwell)

**BRUENING, G. E.** et al.: Biochemical experiments. (Wiley)

**BUSTAMANTE GURRÍA, A.** ed.: Otorhinolaryngology '70 (proc. 9th Internat. Congr.) (Excerpta Medica)

**CHAMPION, R. H.** et al., eds.: An introduction to the biology of the skin. (Blackwell)

**CIBA FOUNDATION:** The pineal gland. (Churchill)

**CIBA FOUNDATION:** Pyrogens and fever. (Churchill)

**CLARK, J. S.:** Group practice. (Livingstone)

**CLARKE, E.:** Modern methods in the history of medicine. (Athlone Press)

**COHEN, E. L.** and **PEGUM, J. S.:** Dermatology. Ed. 2. (Baillière)

**CYRIAX, J.:** Cervical spondylosis. (Butterworth)

**CYRIAX, J.:** The slipped disc. (Gower Press)

**D'ABREU, A. L.** et al.: A practice of thoracic surgery. Ed. 3. (Ed. Arnold)

**DEBRÉ, R.** and **CELERS, J.** eds.: Clinical virology. (Saunders)

**DEBRUNNER, H. U.:** Orthopaedic diagnosis. (Livingstone)

**DE HAAS, J. H.** et al., eds.: Ischaemic heart disease. (Leiden Univ. Press)

**DePALMA, A. F.** and **ROTHMAN, R. H.:** The intervertebral disc. (Saunders)

**DIBLE, J. H.:** Napoleon's surgeon. (Heinemann)

**DOPSON, L.:** The changing scene in general practice. (Johnson)

**DOWBEN, R. M.:** General physiology: a molecular approach. (Harper & Row)

**ELMSLIE, R. G.** and **LUDBROOK, J.:** An introduction to surgery: 100 topics. (Heinemann)

**EVERETT, H. S.** and **RIDLEY, J. H.:** Female urology. (Harper & Row)

**FARNDALE, W. A.:** Law on human transplants. (White's)

**FIELDS, W. S.** and **SPENCER, W. A.** eds.: Stroke rehabilitation. (Green)

**FISHER, J. W.** ed.: Kidney hormones. (Acad. Press)

**FOSTER, W. D.:** A history of medical bacteriology and immunology. (Heinemann)

**FOWLER, N. O.:** Cardiac diagnosis. (Harper & Row)

**FOX, S. A.:** Ophthlamic plastic surgery. Ed. 4. (Grune and Stratton)

**FRIEDMAN, L. J.:** Virgin wives. (Tavistock)

**FRITSCHI, E. P.:** Reconstructive surgery in leprosy. (Wright). Presented by John Wright & Sons Ltd.

**GAMSTORP, I.:** Pediatric neurology. (Butterworth)

**GELIN, L. E.** et al.: Abdominal pain. (Pitman)

**GOLDEN, R.:** Diagnostic radiology, ed. by L. L. Robbins. Section 19: Mammography and breast diseases, by R. J. Egan. (Williams & Wilkins)

**GRABER, C. D.** ed.: Rapid diagnostic methods in medical microbiology. (Williams & Wilkins)

**GREENGARD, P.** and **COSTA, E.** eds.: Role of cyclic AMP in cell function (North-Holland)

**HALLBERG, L.** et al.: Iron deficiency. (Acad. Press)

**HERRLINGER, R.:** History of medical illustration, from antiquity to A.D. 1600. (Pitman)

**HUGO, W. B.:** Inhibition and destruction of the microbial cell. (Acad. Press)

**JAY, J. M.:** Modern food microbiology. (Van Nostrand)

**JOHNSON, S. A.** ed.: The circulating platelet (Acad. Press)

**JOHNSON, S. L.:** History of cardiac surgery, 1896-1955. (Johns Hopkins Pr.)

**KAPPERS, J. A.** ed.: Neurohormones and neurohumors. (Springer)

**KERR, D. N. S.** and **DOUGLAS, A.:** A short textbook of kidney disease. (Pitman)

**KOOS, W. T.** and **MILLER, M. H.:** Intracranial tumours of infants and children. (Thieme/Churchill)

**KROVETZ, L. J.** et al.: Handbook of pediatric cardiology. (Harper & Row)

**LAIDLAW, A. J.** ed.: Outlines of general practice. Ed. 3. (Livingstone)

**LEES, F.:** The diagnosis and treatment of diseases affecting the nervous system. 2 vols. (Staples)

**LEHNINGER, A. L.:** Biochemistry: the molecular basis of cell structure and function. (Worth)

**LITTLE, J. M.:** The management of liver injuries. (Livingstone)

**LLEWELLYN-JONES, D.:** Fundamentals of obstetrics and gynaecology. 2 vols. (Faber)

**MACDONALD, R. R.** ed.: Scientific basis of obstetrics and gynaecology. (Churchill)

**McGREGOR, R. M.:** The work of a family doctor. (Livingstone)

**MANN, F.:** Acupuncture. (Heinemann)

**MARTIN-DOYLE, J. L. C.:** A synopsis of ophthalmology. Ed. 4. (Wright). Presented by John Wright & Sons Ltd.

**MATTHEW, H.** ed.: Acute barbiturate poisoining (Excerpta Medica)

**MENELAUS, M. B.:** The orthopaedic management of spina bifida cystica. (Livingstone)

**MONNIER, M.:** Functions of the nervous system. 2 vols. (Elsevier)

**MORRELL, D. C.:** The art of general practice. (Livingstone)

**MURRAY, R. O.** and **JACOBSON, H. G.:** Radiology of skeletal disorders: exercises in diagnosis. (Livingstone)

**MUSTARDÉ, J. C.:** Plastic surgery in infancy and childhood. (Livingstone)

**NEWELL, F. W.:** Ophthalmology: principles and concepts. Ed. 2. (Mosby)

**NEZLIN, R. S.:** Biochemistry of antibodies. (Plenum)

**O'MALLEY, C. D.** ed.: The history of medical education. (California Univ. Pr.)

**PAUL, J. R.:** History of poliomyelitis. (Yale Univ. Pr.)

**PILLSBURY, D. M.:** A manual of dermatology. (Saunders)

**POLGAR, G.** and **PROMADHAT, V.:** Pulmonary function testing in children. (Saunders)

**PRESTON, F. W.** and **BEAL, J. M.:** Basic surgical physiology. (Year Book)

**QUICK, A. J.:** Bleeding problems in clinical medicine. (Saunders)

**RAFFENSBERGER, J. G.** et al., eds.: The acute abdomen in infancy and childhood. (Lippincott/Blackwell)

**RAMOT, B.:** Clinical aspects of red cell structure and metabolism. (Acad. Press)

**RHOADS, J. E.** et al.: Surgery: principles and practice. Ed. 4. (Lippincott)

**ROBINSON, D. W.:** Occupational hearing loss. (Acad. Pr.)

**RUSSELL, D. S.** and **RUBINSTEIN, L. J.:** Pathology of tumours of the nervous system. Ed. 3. (Ed. Arnold)

**SACKS, O. W.:** Migraine. (Faber)

**SCHMITT, F. O.** ed.: The neurosciences: second study program. (Rockefeller Univ. Pr.)

**SCHNEEBERG, N.:** Essentials of clinical endocrinology. (Mosby/Kimpton)

**SEARCY, R. L.:** Diagnostic biochemistry. (McGraw-Hill)

**SIMMONS, S. C.** and **LUCK, R. J.:** General surgery in gynaecological practice. (Blackwell)

**SLATER, J. D. H.** ed.: Sixth symposium on advanced medicine. (Pitman)

**SOBER, H. A.** ed.: Handbook of biochemistry. Ed. 2. (Chemical Rubber)

**SOLOMON, J. B.:** Foetal and neonatal immunology. (North-Holland)

**SPIRO, H. M.:** Clinical gastroenterology. (Collier-Macmillan)

**SQUIRE, L. F.** et al.: Exercises in diagnostic radiology. 2 vols. (Saunders)

**STEERE, N. V.** ed.: Handbook of laboratory safety. Ed. 2. (Chemical Rubber)

**SYKES, M. K.** et al.: Respiratory failure. (Blackwell)

**THORNE, C.:** Better medical writing. (Pitman)

**WAIN, H.:** History of preventive medicine. (Thomas)

**WALTON, J. N.:** Essentials of neurology. Ed. 3. (Pitman)

**WHITTERIDGE, G.:** William Harvey and the circulation of the blood. (Macdonald)

**WILKINSON, M.:** Cervical spondylosis. Ed. 2. (Heinemann)

**WOODROW, J. C.:** Rh immunisation and its prevention. (Munksgaard)

**YOKOCHI, C.:** Photographic anatomy of the human body. (Igaku Shoin)

**ZOHMAN, L. R.** and **TOBIS, J. S.:** Cardiac rehabilitation. (Grune)

### INDEX TO VOLUME 86

### SUBJECTS

Anticoagulant control — which test? ... ... ... ... ... ... ... ... 71

Association of Clinical Pathologists ... ... ... ... ... ... ... ... 39

Beethoven ... ... ... ... ... ... ... ... ... ... ... ... 43

Bristol Medico-Chirurgical Society ... ... ... ... ... ... ... ... ... 17, 39, 62, 76

New Rules ... ... ... ... ... ... ... ... ... ... 78

Carpal tunnel decompression ... ... ... ... ... ... ... ... ... 33

Dermatoglyphics ... ... ... ... ... ... ... ... ... ... ... 19

Examinations, using multiple choice questions ... ... ... ... ... ... 3

Fracture of tibia, compression plating for ... ... ... ... ... ... ... ... 67

Library, additions to ... ... ... ... ... ... ... ... ... ... ... 15, 41, 64, 80

Maps, west country ... ... ... ... ... ... ... ... ... ... ... 51

Mentally retarded—assessment unit ... ... ... ... ... ... ... ... 27

News and notes ... ... ... ... ... ... ... ... ... ... ... 42, 66

Numerical data, reliability of ... ... ... ... ... ... ... ... ... 7

Obituaries—Alexander, G. L. ... ... ... ... ... ... ... ... ... 37

Golding, H. M. ... ... ... ... ... ... ... ... ... ... 11

Gorham, A. P. ... ... ... ... ... ... ... ... ... ... 61

Roberts, A. E. S. ... ... ... ... ... ... ... ... ... 75

Population explosion ... ... ... ... ... ... ... ... ... ... ... 59

South-west Orthopaedic Club ... ... ... ... ... ... ... ... ... 14, 40, 79

Wisdom from the Past ... ... ... ... ... ... ... ... ... ... 13, 38, 63, 74

### AUTHORS

Briggs, J. C. ... ... ... ... ... 59

Buxton, R. St.J. ... ... ... ... ... 3

Corner, Beryl D. ... ... ... ... ... 43

David, T. J. ... ... ... ... ... 19

Hamblin, T. J. ... ... ... ... ... 71

Jancar, J. ... ... ... ... ... ... 27

Michelinakis, E. ... ... ... ... ... 67

Mitchell, J. P. ... ... ... ... ... 51

Raper, A. B. ... ... ... ... ... 7

Woodyard, J. E. ... ... ... ... ... 33

